# Understanding Later-Life Planning in Singapore: Profiles, Social Determinants, and Policy Insights

**DOI:** 10.1093/geroni/igaf122.2479

**Published:** 2025-12-31

**Authors:** Bussarawan Teerawichitchainan, Biying Yang

**Affiliations:** National University of Singapore, Singapore, Singapore; National University of Singapore, Singapore, Singapore

## Abstract

Planning for estate, long-term care (LTC), and advance care planning (ACP) is a crucial step in successful aging. However, most studies examine financial, LTC, and ACP preparedness separately, despite their interdependence. Additionally, prior research has largely focused on Western contexts, where individual autonomy and well-developed welfare states shape aging experiences. Less is known about later-life estate and healthcare planning in non-Western societies, where norms of family-based support and intergenerational dependence are deeply embedded. Singapore presents a globally relevant case due to its rapid population aging, strong filial piety norms, and proactive government initiatives in later-life planning. The country’s centralized, mandatory, and multi-pillar approach integrates financial security, healthcare, housing, and social support, making it a global leader in structured aging policies. Using latent class analysis on a 2022 nationwide survey of 1,000 parents aged 50+, we identify four distinct profiles of later-life planning: comprehensive planners (9%), estate and LTC planners (31%), basic planners (32%), and the underprepared (28%). Furthermore, results from multinomial logistic regressions reveal socioeconomic inequalities, with higher socioeconomic status, Chinese ethnicity, and native-born individuals being significantly less likely to be underprepared. While marital satisfaction fosters preparedness, number of children is inversely related to planning, and social connectedness with friends and community enhances later-life preparedness. By highlighting Singapore’s distinctive aging policies, our findings expand dimensions of successful aging and underscores the importance of integrated policy approaches that promote later-life preparedness across financial, LTC, and ACP domains.

